# Determination of the infectious titer and virulence of an original US porcine epidemic diarrhea virus PC22A strain

**DOI:** 10.1186/s13567-015-0249-1

**Published:** 2015-09-25

**Authors:** Xinsheng Liu, Chun-Ming Lin, Thavamathi Annamalai, Xiang Gao, Zhongyan Lu, Malak A Esseili, Kwonil Jung, Mohamed El-Tholoth, Linda J Saif, Qiuhong Wang

**Affiliations:** Food Animal Health Research Program, Ohio Agricultural Research and Development Center, College of Food, Agricultural and Environmental Sciences, Department of Veterinary Preventive Medicine, The Ohio State University, Wooster, Ohio USA; State Key Laboratory of Veterinary Etiological Biology, OIE/National Foot-and-Mouth Disease Reference Laboratory of China, Lanzhou Veterinary Research Institute, Chinese Academy of Agricultural Sciences, Lanzhou, China; Department of Virology, Faculty of Veterinary Medicine, Mansoura University, Mansoura, Egypt

## Abstract

The infectious dose of a virus pool of original US PEDV strain PC22A was determined in 4-day-old, cesarean-derived, colostrum-deprived (CDCD) piglets. The median pig diarrhea dose (PDD_50_) of the virus pool was determined as 7.35 log_10_ PDD_50_/mL, similar to the cell culture infectious titer, 7.75 log_10_ plaque-forming units (PFU)/mL. 100 PDD_50_ caused watery diarrhea in all conventional suckling piglets (*n* = 12) derived from a PEDV-naive sow, whereas 1000 and 10 000 PDD_50_ did not cause diarrhea in piglets derived from two PEDV-field exposed-recovered sows. This information is important for future PEDV challenge studies and validation of PEDV vaccines.

## Introduction, methods, and results

Porcine epidemic diarrhea (PED) is a highly contagious enteric disease of swine characterized by acute watery diarrhea, vomiting, dehydration, and weight loss. It was first observed in farm pigs in England in 1971 [1]. The causative agent, PED virus (PEDV), was identified in 1978 [2]. Subsequently, PEDV caused epidemics with pig mortality in European countries until the late 1980s. Thereafter PEDV was more often associated with endemic cases in Europe. In Asia, epidemics with major losses in suckling pigs were first reported in 1982, continuing into the 1990s-2000s [3-5]. Since October 2010, severe PED epizootic outbreaks, affecting pigs of all ages but characterized by high mortality rates among suckling piglets, have been reported in China, causing significant economic losses [6]. In April 2013, PEDV emerged in US swine and spread rapidly throughout the country, leading to the death of 8 million pigs and economic losses between $900 million - $1.8 billion in 2013–2014 [7,8].

The pathogenesis of the original US PEDV isolates has been evaluated in gnotobiotic (Gn) [9], cesarean-derived, colostrum-deprived (CDCD) [10], and conventional pigs [11]. In agreement with field observations, the original US PEDV isolates were highly virulent with 100% morbidity and 50-100% mortality in suckling piglets [12]. Intentional exposure of pregnant sows to PEDV via feedback of fecal material or/and the intestinal tracts of infected piglets stimulated maternal immunity and shorted the outbreaks on some farms [8]. However, outbreaks of PEDV continue in the US. The development of effective vaccines is urgently needed to prevent PED.

To evaluate vaccine efficiency in vivo, challenge of piglets derived from immunized sows with a standardized and validated dose of PEDV is required. To date, information on the infectious titer of a PEDV challenge pool is limited and a standard PEDV inoculum is needed to assess PEDV vaccine efficacy. In this study, the median pig diarrhea dose (PDD_50_) of an original US PEDV, PC22A strain, was determined by using 4-day-old CDCD piglets and was confirmed using conventional suckling pigs. Also, the acute clinical signs and pathological lesions were studied. The animal use protocols were reviewed and approved by the Agricultural Animal Care and Use Committee, The Ohio State University.

Four PEDV-naïve sows (A, B, C, D) with no previous herd history of PED outbreaks and that tested PEDV seronegative (Veterinary Diagnostic Laboratory, University of Minnesota (UMN)) were identified. They were subjected to cesarean section for the derivation of CDCD pigs (*n* = 53). On the day of birth, the piglets were fed bovine colostrum replacer (AgriLabs, St. Joseph, MO, USA) that was gradually replaced by whole milk (Parmalat, Parmalat USA Corp) by the third day. Treatments with antibiotics (Penicillin (VET one, UK), gentamicin (VET one, UK) and neomycin (MED-PHARMEX, Pomona, CA, USA)), antitoxin (Clostridium antitoxin (Boehringer Ingelheim Vetmedica, St. Joseph)) and probiotics (probios (Chr. Hansen, Menomonie, WI, USA)), as prescribed by the clinician, were performed to prevent opportunistic infections of piglets throughout the experiment. All piglets were randomly allocated into 8 dose-groups (G1-G8) and two mock control groups (Table 1). Each piglet was housed in individual steel cages and the same group of pigs was in cages installed in 3 levels (2–4 cages/level) above the floor. The cages were maintained in biological safety level II (BSL2) rooms. Two adjacent dose-groups of pigs were housed in different sides of the same room (5.4 × 3.0 m^2^).

**Table 1:** **Summary of pig groups and corresponding numbers, inoculum and pig diarrhea outcomes after inoculation**

**Pig Group**	**Pig numbers**	**Inoculum dilution** ^**a**^	**Calculated inoculum infectious titers (log**_**10**_ **PFU/mL)** ^**b**^	**Calculated inoculum RNA titers (log**_**10**_ **GE/mL)** ^**b**^	**Diarrhea (percent)** ^**c**^	**Fecal virus RNA shedding (log**_**10**_ **GE/mL)**
G1	4	10^−3^	4	10	4/4 (100)	11.8-13.0
G2	5	10^−4^	3	9	5/5 (100)	12.5-12.9
G3	5	10^−5^	2	8	5/5 (100)	12.0-13.4
G4	13	10^−6^	1	7	13/13 (100)	9.8-13.3
G5	8	10^−7^	0	6	8/8 (100)	11.5-13.7
G6	5	10^−8^	−1	5	2/5 (40)	11.4-12.4
G7	4	10^−9^	−2	4	0/4 (0)	-^d^
G8	5	10^−10^	−3	3	0/5 (0)	-
Control 1^g^	2	PBS	-	-	0/2 (0)	- or 5.0
Control 2	2	PBS	-	-	0/2 (0)	-
One litter of PEDV-naïve Sow E	12	10^−5^(100 PDD_50_)	2	8	12/12 (100)	11.8-13.0
One litter of PEDV-preexposed Sow F	13	10^−3^ (10,000 PDD_50_)	4	10	0/13 (0)	5.1-8.9 ^e^
One litter of PEDV-preexposed Sow G	10	10^−4^ (1,000 PDD_50_)	3	9	0/10 (0)	4.9-5.5 ^f^

^a^Each pig was inoculated orally with 3 mL inoculum.

^b^Titers were calculated based on the titer of the original virus pool and dilution times.

^c^Fecal scores of 3 as determined by 24 hpi for pigs in G1-G8 and control groups and conventional pigs of sow E, and through 7 and 9 dpi for conventional pigs of sows F and G, respectively.

^d^Negative or below detection limit of RT-qPCR (4.8 log_10_GE/mL).

^e^Data of 10 pigs, which tested positive.

^f^Data of 2 pigs, which tested positive.

^g^Piglets of control 1 group were housed in the same room as G5 piglets.

The virus pool of PEDV PC22A strain was prepared in a gnotobiotic (Gn) pig as described in our previous study [9]. The second passage (P2) of tissue culture-adapted PC22A [13] was plaque-purified and one plaque was selected and propagated once more (total P3) in Vero cells. It was used as inoculum (5 log_10_ plaque-forming unit (PFU)/mL) to orally challenge a 19-day-old Gn pig. The pig was euthanized at 1 day post-inoculation (dpi) when it had watery diarrhea. The small and large intestinal contents were collected at 1 dpi aspectically, mixed and stored in 1 mL aliquots at −80 °C as the virus pool. The infectious and RNA titers of the virus pool were 7.75 log_10_ PFU/mL and 13 log_10_ genomic equivalents (GE)/mL, respectively, using plaque assay and quantitative real-time reverse transcription-PCR (RT-qPCR) as reported [13]. For the preparation of inocula for pigs, 1 mL of PC22A virus pool was diluted 1:10 in phosphate-buffered saline (PBS, pH 7.4; Sigma-Aldrich, St. Louis, MO), mixed well, and vortexed. The suspension was centrifuged at 2095 × *g* for 10 min at 4 °C, and the supernatant was collected as the 1:10 (10^−1^) dilution. Subsequently, 10-fold serial dilutions (10^−2^-10^−10^) were prepared in PBS.

Each experimental group (G1-G8) of piglets was inoculated orally with 3 mL of the 10-fold serially diluted (10^−3^-10^−10^) virus at 4 days of age. The control groups received PBS (Table 1). After inoculation, piglets were observed 4 times daily for clinical signs, including diarrhea. Rectal swabs were collected daily from all piglets and scored for fecal consistency: 0 = normal; 1 = pasty; 2 = semi-liquid; and 3 = liquid. Scores of 3 were considered as watery diarrhea. Median pig diarrhea dose (PDD_50_) was determined as the reciprocal of the virus dilution at which 50% of the pigs developed watery diarrhea at a given time point using the Reed and Muench method [14].

To reduce the risk of cross contamination among pigs, PEDV PC22A-inoculated CDCD piglets were euthanized at onset of watery diarrhea and subjected to necropsy examination. Duodenum, jejunum, ileum, cecum, colon and mesenteric lymph nodes were collected and fixed in 10% neutral buffered formalin for histopathological examinations as described previously [9]. For each jejunum section, ten villi and crypts were measured using a computerized image system (PAX-it software, PAXcam, Villa Park, IL, USA) [9]. Villous height and crypt depth ratios (VH:CD) were calculated. Also, PEDV nucleocapsid (N) proteins were detected by immunohistochemistry (IHC) using mouse monoclonal antibody (SD6-29) (gift from Drs. Steven Lawson and Eric Nelson at South Dakota State University) [15].

Because conventional suckling pigs are the targets for future vaccine studies, PEDV-naïve sow E was selected and the naturally delivered suckling piglets were inoculated orally with PC22A at 100 PDD_50_/pig at 4 days of age to verify the results from the CDCD pig experiments.

In addition, two PEDV-field exposed-recovered sows F and G were obtained from a farm with a recent PEDV outbreak (July 19, 2014) and subsequent exposure to live virus for 3 continuous days (July 20–22, 2014), at 73 to 75 days pre-farrowing. Serum samples of sows F and G tested positive for PEDV-specific IgG, IgA and virus neutralizing antibodies at 53 days post-outbreak (20 and 22 days pre-farrowing) (Table 2) by PEDV-specific cell culture immunofluorescence (CCIF) and plaque reduction virus neutralization (PRVN) assays as described [16]. Sows F and G delivered 13 and 10 piglets, respectively, by natural farrowing. Piglets and their sows were housed together in separate rooms for each litter. At 4 days of age, piglets of sow F and G were inoculated orally with 10 000 PDD_50_ and 1000 PDD_50_, respectively. On 7 dpi and 9 dpi, respectively, the piglets and their sows were euthanized.

**Table 2:** **PEDV-specific IgG, IgA and virus neutralizing (VN) antibodies of serum and milk samples of PEDV field exposed sows F and G**

**Sample tested**		**Pre-farrowing** ^**a**^		**After piglet inoculation** ^**a**^	
		**Sow F**	**Sow G**	**Sow F**	**Sow G**
Serum	IgG	128	64	128	128
	IgA	32	32	8	16
	VN	147	222	128	128
Milk	IgG	NA ^b^	NA	1024	1024
	IgA	NA	NA	512	128
	VN	NA	NA	1351	675

^a^Serum of sows F and G were collected at 20- and 22-days pre-farrowing and at 7 or 9 day post-inoculation (dpi), respectively. Piglets of Sow F and G were inoculated with 10 000 and 1000 PDD_50_, respectively, at 4 days of age. Milk samples of sows F and G were collected at 1 dpi.

^b^NA: not applicable.

Porcine epidemic diarrhea virus RNA fecal shedding in rectal swab samples or intestinal contents was negative before virus inoculation and became positive on 1 dpi in G1-G6 CDCD piglets (10^−3^-10^−8^ diluted virus), with titers ranging from 9.8-13.7 log_10_ GE/mL (Table 1). By 1 dpi, 100% of pigs of G1 to G5 and 40% (2/5) of G6 had diarrhea, and no pigs in G7 and G8 (10^−9^ and 10^−10^ diluted virus) and control groups 1 and 2 had diarrhea. The 2 pigs in control group 1 were housed in the same room as G5 pigs (with 10^−7^ diluted virus). They were clinically healthy on 1 dpi but developed watery diarrhea on 2 dpi. The two pigs of control group 1 shed viral RNA on 1 and 2 dpi, respectively. These results indicated that cross contamination of PEDV occurred between the two groups (G5 and control 1) of pigs housed in the same room. The cut-off time point was set as 1 dpi for determination of the PDD_50_ which was 7.83 PDD_50_/3 mL, corresponding to 7.35 log_10_ PDD_50_/mL. It was similar to the cell culture infectious titer (7.75 PFU/mL) determined by plaque assay. No clinical signs were observed and no PEDV RNA shedding was detected by 3 dpi in the two pigs of control 2 group.

**Figure 1:**
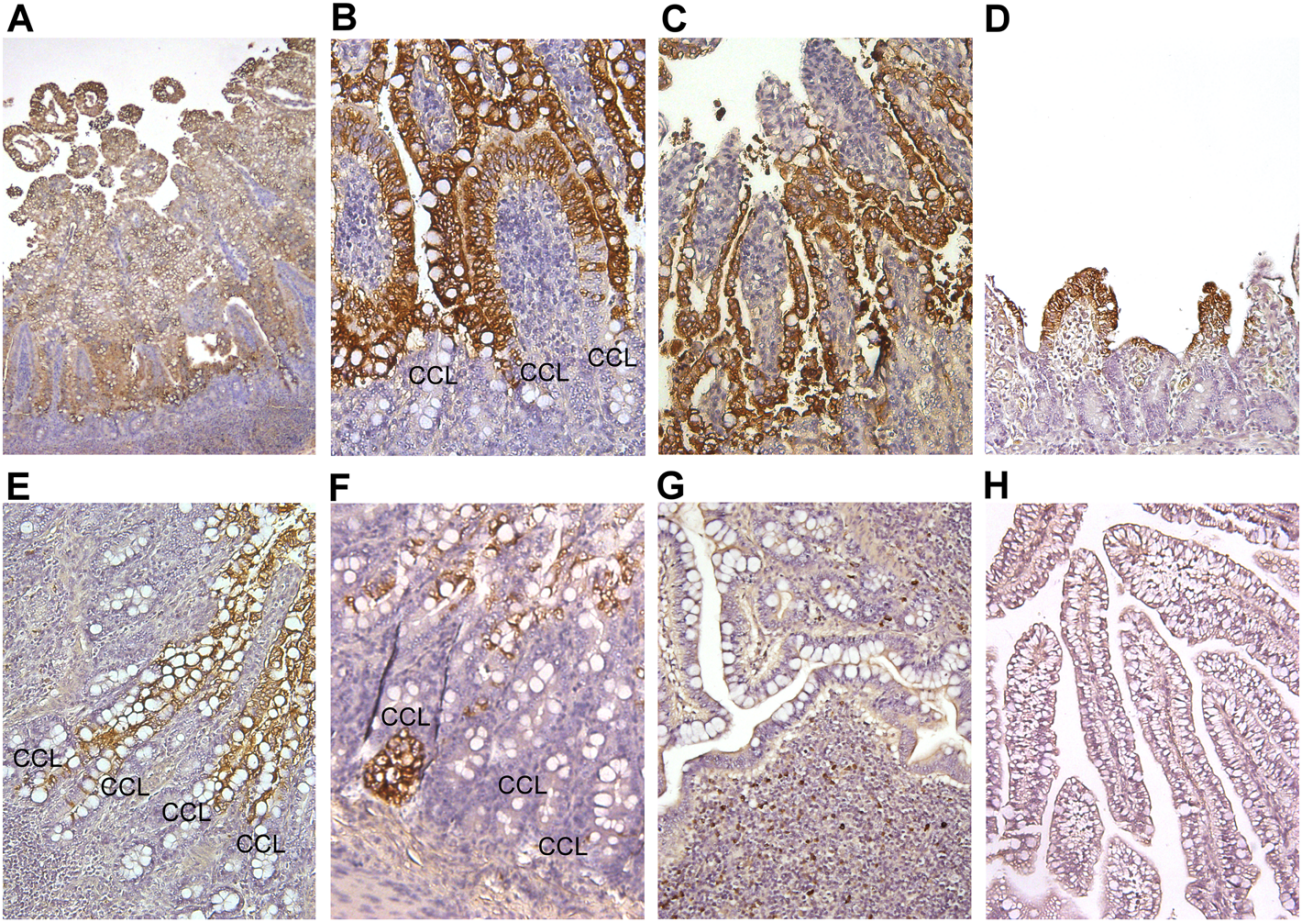
**Detection of PEDV PC22A infection by immunohistochemistry (IHC) staining using mouse anti-PEDV nucleocapsid monoclonal antibody (SD6-29).** Signal of PEDV antigens were in brown color and detected in the entire villous epithelial cells in the middle jejunum of PEDV PC22A-inoculated CDCD piglets **(A**-**D)**. Shortening and fusion of villi along with exfoliation of enterocytes were observed **(C, D)**. Depending on the progress of PEDV infection, PEDV-infected villous epithelial cells still formed villi **(B)**, detaching, swelling and undergoing necrosis **(C)**, or highly attenuated **(D)**. PEDV antigens extended to the villus/crypt border and were sporadically located in the crypt cell layer (CCL) in ileum obtained from CDCD **(E)** and conventional **(F)** piglets. The majority of the residual epithelial cells were PEDV-positive. In addition, PEDV antigens were detected in few mononuclear cells in dome area in ileum obtained from conventional, PEDV-inoculated piglets derived from two PEDV-exposed and recovered sows **(G)**. No PEDV antigen was detected in the jejunum of a mock-inoculated CDCD piglet **(H)**. Original magnifications: × 40 **(A)**, × 200 **(B-H)**.

Microscopically, different stages of villous atrophy (Figures 1A-D) were observed in the same group of CDCD piglets receiving the same virus dose at 1–3 dpi. In some piglets, the length of villi decreased slightly and the morphology of villous epithelial cells was generally intact (Figures 1A-B). In other cases, significant shortening of villi along with exfoliation and vacuolation of enterocytes were observed (Figure 1C). As the severity of villous atrophy increased, the villi were blunted and fused throughout the entire small intestine (Figure 1D). However, there were no significant differences in the degree of villous atrophy, and PEDV antigen scores among pig groups receiving different doses. Overall, the majority (40/49, 82%) of PEDV-inoculated CDCD piglets had mean jejunum VH:CD ratios <2. In contrast, the mean jejunum VH:CD ratios of the 2 control pigs of control group 2 were 7.16 ± 1.25 and 7.80 ± 0.60, respectively.

No PEDV N proteins were detected in tissues collected from the 2 pigs of control group 2 by IHC (Figure 1H). However, for the PEDV-inoculated pigs, the entire small intestine was positive for PEDV N proteins, which were detected mainly in the cytoplasm of enterocytes located both on the tip and lateral walls of the villi extending to the villus/crypt border (Figures 1A-B). Less frequently, PEDV-positive cells were observed in or near the crypts of Lieberkühn (5/49, 10%) (Figures 1A-B). The highest IHC signal intensity was detected in some cases with relatively higher VH:CD ratios (2.54 to 5.17) (Figures 1A-C). As the severity of villous atrophy increased, the total number of villous epithelial cells and PEDV-positive cells decreased dramatically (Figure 1D). Scattered foci of PEDV antigens were detected in the colonic epithelia in 6/49 (12%) PEDV-inoculated CDCD pigs (data not shown).

The 12 suckling piglets of PEDV-naive sow E had watery diarrhea on 1 dpi with a high level of PEDV RNA shedding (11.8-13.0 log_10_ GE/mL). The piglets that died or were euthanized between 1 and 5 dpi had mean VH:CD ratios ranging from 0.48 ± 0.12 to 1.57 ± 0.32, except for one piglet that was euthanized at 5 dpi that showed a relatively higher mean VH:CD ratio (2.29 ± 0.64). Patterns of PEDV antigen distribution were similar to those observed in CDCD piglets (data not shown). Degeneration of crypt epithelial cells containing abundant PEDV N antigen was noted in one (1/8) conventional piglet euthanized at 5 dpi (Figure 1F).

The piglets of sows F and G did not have diarrhea by 7 dpi and 9 dpi, respectively. Some piglets of sows F (3/13, 23%) and G (8/10, 80%) did not shed PEDV RNA in feces, and the remaining piglets shed virus at low titers (4.9-8.9 log_10_ GE/mL) (Table 1). The serum samples of sows F and G collected at euthanasia (7 or 9 dpi) had similar PEDV-specific antibody titers as the pre-farrowing titers, consistent with lack of PEDV re-stimulation of the sows due to piglet protection (Table 2). Milk samples of sow F and G collected on 1 dpi had much higher PEDV-specific antibody titers (1024 (IgG), 512 (IgA) and 1351 (VN) for Sow F and 1024 (IgG), 128 (IgA) and 675 (VN) for sow G, respectively) than serum samples (Table 2).

On 7 or 9 dpi, no significant histopathological lesions were observed in piglets of sows F and G. PEDV N proteins were detected in individual mononuclear cells in intestinal submucosa/Peyer’s patches but not in intestinal villous epithelial cells (Figure 1G).

## Discussion

In this study, we used CDCD pigs to determine the PDD_50_ of a PEDV virus pool for several reasons: 1) Random allocation of CDCD piglets into different groups eliminated the influence of litter-to-litter variation; and 2) to reduce the number of experimental animals required for the determination of PDD_50_, compared to using conventional suckling pigs in which at least one litter of pigs is required per group. In this study, 40% (2/5) of piglets of G6 (with 10^−8^ diluted virus, calculated dose of 0.3 PFU per pig) showed severe diarrhea by 1 dpi. After the PDD_50_ was determined in CDCD pigs, the results were confirmed in age-matched conventional pigs, suggesting CDCD piglets are a reliable and economic model to determine the PDD_50_ and virulence of a PEDV strain.

Recently, airborne transmission of PEDV was confirmed experimentally in some [17], but not in other studies [18]. One limitation of the present study was that the possibility of PEDV airborne transmission could not be excluded completely since no separate air handling system was available for the control group 1 cages and the inoculated group 5 cages. Our results indicated that even low dose PEDV inoculum resulted in high titers (>10 log_10_GE/mL) of fecal PEDV shedding in piglets. High levels of PEDV in feces from the first breaking pigs may increase the risk of cross-contamination. Two pigs of control group 1 (Table 1) became infected at 24–48 hours post-inoculation (hpi) possibly via airborne transmission of PEDV from the inoculated G5 piglets (10^−7^ dose), which were housed on the other side of the same room. Because of this concern, we decided to set the cut-off time point as 24 hpi for determination of the PDD_50._ Also the additional control piglets were maintained in a separate room and tested negative for PEDV. Piglets (G6-G8) receiving very low doses, calculated <1 PFU, still had diarrhea, but after 1 dpi. Therefore, the actual minimal infectious dose could be lower than 1 PFU. Our data suggest that piglet bioassay is more sensitive to PEDV infection than Vero cells. This may explain why it is so difficult to eliminate PEDV endemic infections from a farm with a history of PEDV outbreaks.

Since the first emergence of prototype PEDV CV777 in England in 1971 [1], different PEDV strains with various genetic [13,19,20] and antigenic [20] properties have evolved or emerged. In this study, dominant clinical signs and microscopic lesions and high fecal viral shedding were observed in PEDV PC22A-inoculated piglets (>1 PFU) within 1 dpi. The VH:CD ratios were less than 2 in the majority of PEDV PC22A-infected CDCD piglets. On the other hand, a longer incubation time (22–36 hpi) was reported in prototype PEDV CV777-inoculated, age-matched piglets (10^4^ pig infectious dose/pig) [21,22]. A previous Korean endemic PEDV strain SNUVR971496 induced severe villous atrophy in younger (1-day-old) colostrum-deprived piglets [23]. However, no PEDV antigen or nucleic acid was detected in epithelial cells within the crypts in PEDV SNUVR971496-infected pigs [23]. It was proposed that current US or US-like PEDV outbreaks are more severe than those previously described in Europe and Korea [3]. Our results suggest that the clinical and histopathological features of PEDV PC22A infection are generally comparable or more severe than those reported for historical European or previous endemic Korean PEDV infection in 1- to 3-day-old piglets. However, this speculation needs to be proven by experimental infection of pigs with historical and emerging PEDV strains under the same condition. In agreement with previous studies that PEDV antigens of CV777 [21,22] and the original US PEDV strain [10,12] were detected in crypt cells, we found that infection of crypt cells by PEDV PC22A strain was observed in about 10% (5/53) of CDCD piglets and in one of 12 conventional sucking piglets. In addition, a strong PEDV antigen signal was detected in crypts (Figure 1). The significance of this finding is unknown.

Our published in vitro study showed that anti-PEDV CV777 convalescent serum cross-reacted with US original PEDV PC22A by CCIF assay for IgG antibodies and by viral neutralization assay. However, the antibody titers against homologous PEDV strains were higher than those against heterologous strains [16]. These results are in agreement with the reported genetic variances between classical/historical PEDV strains, including the prototype PEDV CV777, and original US PEDV strains [19]. Similarly, testing of antigenic cross-reactivity between three current Korean field PEDV strains and two vaccine strains (SM98, P5-V) also revealed antigenic variation between field and vaccine PEDV strains [20]. Current Asian (Chinese, Thailand, Japanese, Vietnam and Korean) PEDV pandemic strains have higher genetic similarity to original US PEDV strains than to classical PEDV strains [8]. These results may explain why the vaccines based on classical PEDV strains did not work well in these countries. Alternatively, it is unclear from published studies if the PEDV vaccine strain used induced sufficient lactogenic immunity in the sows to protect their nursing piglets against high challenge doses of virulent PEDV strains. In this study, the two field exposed PEDV-recovered sows F and G were obtained from a farm with a known history of a recent PEDV outbreak and immediate exposure to live virus for 3 continuous days at 73–75 days pre-farrowing. It is most likely that these sows were exposed to homologous or genetically similar original US PEDV strains. Since feedback was performed on the farm for 3 continuous days and within 2–3 months before farrowing, the sows had lactogenic immunity (documented in Table 2). Therefore, the suckling piglets of sows F and G were most likely protected from PEDV diarrhea after challenge with high doses (1000 and 10 000 PDD_50_) of virulent PEDV which were 10–100 times higher than the 100 PDD_50_ that caused 100% diarrhea in piglets derived from PEDV-naïve sow E. The piglets were likely protected by PEDV-specific antibodies detected in the milk of PEDV-recovered sows (Table 2). In addition, no booster antibody response was detected in serum samples of sows F and G, likely because all piglets were protected from disease and only some pigs shed PEDV at low levels after PEDV-inoculation.

In conclusion, the PDD_50_ of a virus pool of original US PEDV PC22A strain was determined. This information is important for future PEDV challenge studies and the validation of PEDV vaccine efficacy. Extremely low infectious dose, short incubation time, severe clinical signs and intestinal lesions and extensive infection of the intestine were characteristics of PEDV PC22A strain infection.

## Abbreviations

CDCD: Cesarean-derived, colostrum-deprived
ELISA: Enzyme-linked immunosorbent assay
HRP: Horseradish peroxidase
IHC: Immunohistochemistry
PDD50: The median pig diarrhea dose
PED: Porcine epidemic diarrhea
PEDV: Porcine epidemic diarrhea virus
PFU: Plaque-forming units
DPI: Day post-inoculation
hpi: Hours post-inoculation
RS: Rectal swab
RT-qPCR: Quantitative real-time reverse transcription-PCR
VH:CD ratios: Villous height and crypt depth ratios
VN: Virus neutralizing/neutralization
